# Next-Generation Vaccines Based on Bacille Calmette–Guérin

**DOI:** 10.3389/fimmu.2018.00121

**Published:** 2018-02-05

**Authors:** Natalie E. Nieuwenhuizen, Stefan H. E. Kaufmann

**Affiliations:** ^1^Max Planck Institute for Infection Biology, Berlin, Germany

**Keywords:** tuberculosis, *Mycobacterium bovis* bacille Calmette–Guérin, vaccine, recombinant *Mycobacterium bovis* bacille Calmette–Guérin, subunit vaccine, mycobacteria

## Abstract

Tuberculosis (TB), caused by the intracellular bacterium *Mycobacterium tuberculosis* (Mtb), remains a major health threat. A live, attenuated mycobacterium known as Bacille Calmette–Guérin (BCG), derived from the causative agent of cattle TB, *Mycobacterium bovis*, has been in clinical use as a vaccine for 90 years. The current incidence of TB demonstrates that BCG fails to protect sufficiently against pulmonary TB, the major disease manifestation and source of dissemination. The protective efficacy of BCG is on average 50% but varies substantially with geographical location and is poorer in those with previous exposure to mycobacteria. BCG can also cause adverse reactions in immunocompromised individuals. However, BCG has contributed to reduced infant TB mortality by protecting against extrapulmonary TB. In addition, BCG has been associated with reduced general childhood mortality by stimulating immune responses. In order to improve the efficacy of BCG, two major strategies have been employed. The first involves the development of recombinant live mycobacterial vaccines with improved efficacy and safety. The second strategy is to boost BCG with subunit vaccines containing Mtb antigens. This article reviews recombinant BCG strains that have been tested against TB in animal models. This includes BCG strains that have been engineered to induce increased immune responses by the insertion of genes for Mtb antigens, mammalian cytokines, or host resistance factors, the insertion of bacterial toxin-derived adjuvants, and the manipulation of bacterial genes in order to increase antigen presentation and immune activation. Subunit vaccines for boosting BCG are also briefly discussed.

## Introduction

The bacterium *Mycobacterium tuberculosis* (Mtb) remains one of the most difficult pathogens to control, and caused 10.4 million recorded cases of tuberculosis (TB) and 1.7 million recorded deaths in 2016 (1). Just under a quarter of the world’s population is estimated to be latently infected with Mtb, with the highest prevalence in Africa and Asia (2). The risk of developing active disease for those with latent TB infection (LTBI) is greatest within the first 2 years and approximately 10% over a lifetime, or 5–10% per year in HIV infected individuals (3). Treatment with drugs requires 6–9 months of antibiotics, and not only multi-drug resistant (MDR) strains but also extremely drug-resistant (XDR) strains continue to emerge, leading to extended drug treatment regimens with considerable side effects (4, 5). The HIV pandemic and socio-economic factors are the two major drivers of TB disease, with factors such as poor living conditions and sanitation, crowded housing, poor air quality, malnutrition, stress, and co-infections all increasing susceptibility to developing active TB disease (6). Improvement of socio-economic conditions along with development of a more effective vaccine against TB will be critical in controlling this devastating disease.

Almost 100 years ago, in 1921, the first newborn was immunized with a live attenuated strain of the bovine *Mycobacterium* species, *Mycobacterium bovis* bacille Calmette–Guérin (BCG), followed by mass vaccination campaigns (7). BCG is partially protective against TB and has immunostimulatory effects that reduce general mortality during the first years of life by enhancing responses to other infectious diseases such as respiratory viruses (8–10). However, the efficacy of BCG against TB varies geographically and BCG does not provide adequate protection against pulmonary disease, the main form of disease manifestation and the cause of transmission (1). The development of a more effective TB vaccine is therefore likely to play a profound role in controlling this disease. As a live vaccine, BCG can also cause local or systemic infection in immunocompromised individuals (11) and is thus contraindicated in individuals who stand to benefit most from vaccination, such as HIV-positive individuals who are at high risk of developing active TB. Hence, the development of a vaccine that is safer for use in immunocompromised individuals is also a high priority.

A number of TB vaccine candidates are under clinical development, and many more have been pre-clinically tested in animal models (12–15). Pre-clinical evaluation of novel vaccine candidates has improved our knowledge of protective responses against TB and has shown that as a standalone vaccine BCG is at least as effective as novel subunit vaccines (16). BCG continues to be used in countries where TB is endemic due to its partial efficacy and has an established safety record. Hence, two major strategies in TB vaccine development have been to generate live mycobacterial vaccines with improved efficacy and safety, such as recombinant BCG (rBCG) vaccines, or to boost BCG with subunit vaccines containing Mtb antigens. This review provides an update on the latest knowledge on BCG and summarizes the rBCG candidates that have been tested against TB in animal models or clinical trials.

## BCG as a Vaccine Against TB

Meta-analyses have found that BCG provides on average 50% protection against TB and is effective for 10–20 years, but efficacy varies between countries and is much lower in adults than in children (17–21). Absence of sensitization to environmental mycobacteria or prior Mtb infection is associated with higher efficacy of BCG against TB (18). BCG is particularly effective against TB meningitis and disseminated TB in infants, with protection against pulmonary TB being much lower (22). The original BCG developed at the Pasteur Institute in Lille, France, was distributed around the world, and continuing passaging led to accumulating genetic mutations and the divergence of numerous substrains (23). These substrains appear to vary in efficacy in animal models, which has been reviewed previously (23). It has been suggested that this could contributes to the variable efficacy seen in different studies; however, a meta-analysis suggests that the type of BCG substrain does not significantly affect efficacy (18). More strikingly, analyses found higher efficacy in colder countries such as UK and Norway and lower efficacy in warmer countries such as India and Indonesia (18, 19, 22, 24, 25). This variation in efficacy seems to be due to increased exposure to environmental mycobacteria, which appears to reduce reactivity to BCG (18, 26, 27). Prior infection with Mtb also reduces the efficacy of the BCG vaccine (18). People living in TB endemic countries are more frequently exposed to Mtb, which raises the risk of individuals being infected (28). The HIV pandemic has contributed to increasing the burden of TB (3). Other risk factors for TB disease include diabetes, smoking, alcoholism, indoor air pollution, chronic corticosteroid treatment, malignancy, and malnourishment (29, 30). Therefore, these factors probably also contribute to the failure of BCG to protect against disease in some individuals.

Humans are not the only species at risk of TB, as wildlife and farmed animals are also susceptible to infection with various mycobacterial strains. Two species of agricultural importance include *M. bovis* and *Mycobacterium caprae*, closely related species of the same clade that cause TB in cattle and goats, and can also be transmitted to humans (31). BCG was first tested and proven effective against virulent *M. bovis* infection in cattle by Calmette and Guerin in 1911 (32), 10 years before its delivery to a human newborn; however, it is not routinely given to cattle to avoid interference with diagnostic tests for *M. bovis* (32). Recently, it has been shown feasible to distinguish vaccinated from infected animals (33, 34). Trials vaccinating large animals with BCG and subunit vaccine boosters have demonstrated that BCG is more protective when administered to neonates and that subunit vaccines can boost protection after BCG prime [reviewed in Ref (32)]. Cows and goats vaccinated with BCG and exposed to natural infection were protected compared to those without vaccination (32). Efficacy was approximately 55–70%, similar to the estimated efficacy of BCG in humans in Norway and the UK (19, 35, 36).

**Figure 1:**
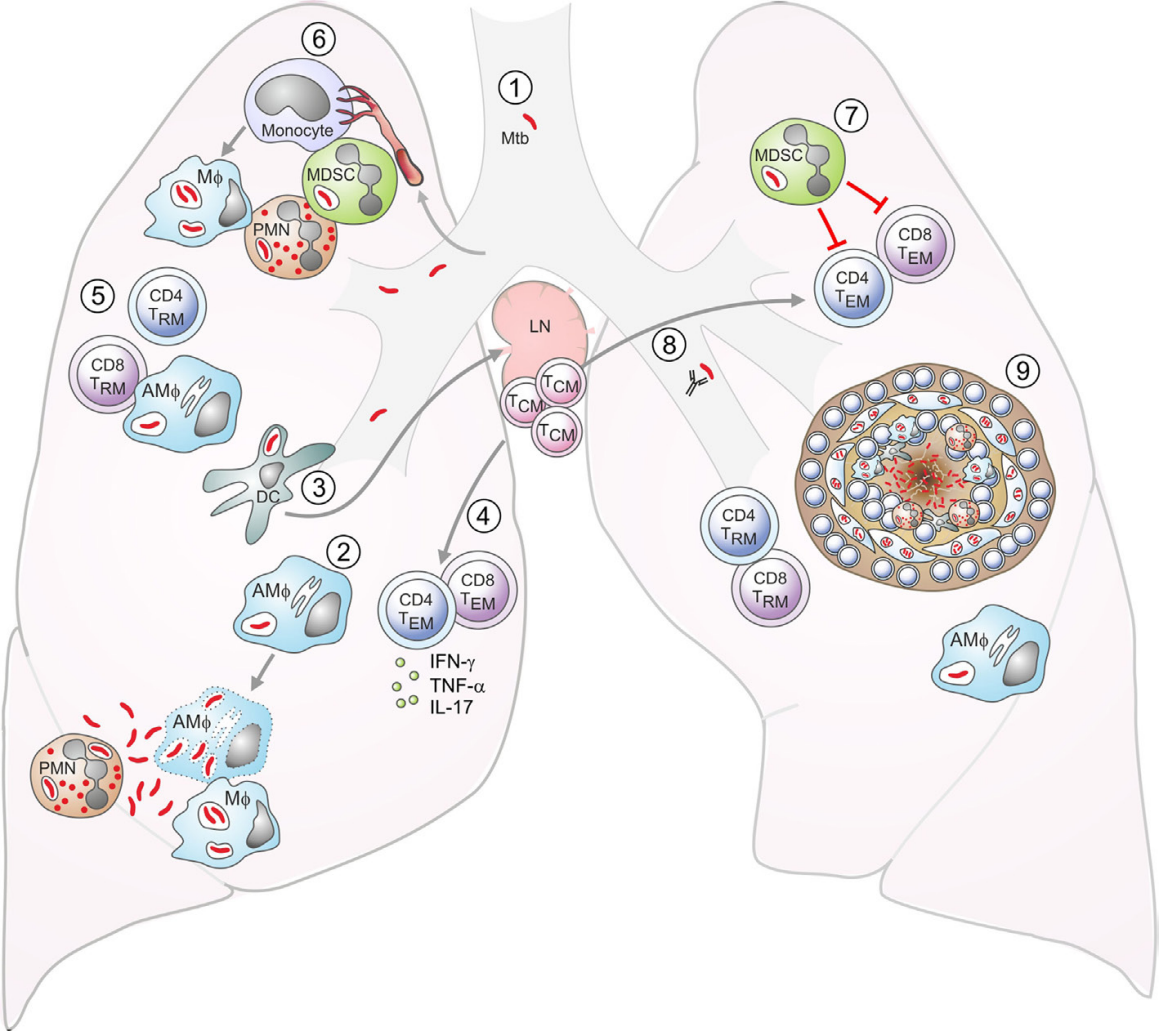
Aspects of tuberculosis (TB) immunology. DC: dendritic cell; Mϕ: macrophage; AMϕ: alveolar macrophage; T_EM_: effector memory T cell; T_CM_: central memory T cell; T_RM_: resident memory T cell; MDSC: myeloid-derived suppressor cell; PMN: neutrophil. (1) Exposure to *Mycobacterium tuberculosis* (Mtb) is by the inhalation of infected aerosol droplets generated by coughing. (2) Mtb bacilli primarily live in host macrophages. Alveolar macrophages and other myeloid cells such as DCs can take up bacteria from the airways. If macrophages do not control infection, the bacteria can replicate and kill the cell. Bacteria can also infect neutrophils, which die and can be taken up by macrophages, which subsequently become infected themselves. (3) Infected DCs have delayed migration to the lymph nodes and impaired antigen presentation. In the lymph nodes, they transfer antigens to uninfected bystander DCs, which present the antigens to T cells. (4) Effector CD4^+^ and CD8^+^ T cells generated from naive T cells or from memory T cells are recruited to the lungs. (5) Effector CD4^+^ T cells produce cytokines such as IFN-γ, interleukin (IL)-17, and TNF-α, while CD8^+^ T cells can lyse infected macrophages. IFN-γ can activate bacterial killing. (6) Neutrophils, monocytes, or immature myeloid cells with suppressive functions, known as MDSCs are recruited to the lungs. Monocytes can differentiate into inflammatory macrophages or inflammatory DCs. (7) MDSCs aim to limit excessive inflammation by inhibiting T-cell proliferation and function, but they can act as a reservoir for Mtb. (8) Antibodies may also play a role in controlling infection. (9) Eventually, the immune cells form a granuloma around Mtb in an attempt to contain the bacteria. These are the typical lesions observed in the Mtb-infected lung, most commonly in the upper lobes.

Vaccines rely on the generation of memory responses, which result from the clonal expansion and differentiation of antigen-specific lymphocytes (37). T cells can differentiate into effector memory T (T_EM_) cells that have effector functions such as cytokine production and migrate to affected tissues, and central memory T (T_CM_) cells that home to secondary lymphoid organs, where they can proliferate and differentiate into new effector cells upon re-exposure to antigen. Immunity to Mtb requires CD4^+^ and CD8^+^ T cells producing type I effector cytokines such as IFN-γ and TNF-α and their recruitment to the lung (38–43). T cells that home to the lung tissue and accumulate there, known as tissue resident memory (T_RM_) cells, are particularly important for immunity to Mtb (38, 43). Mucosal immunization with BCG induces more T_RM_ cells than parental immunization (43). In addition, it was recently shown that interleukin (IL)-21-dependent memory-like natural killer (NK) cells generated after BCG vaccination were protective against Mtb challenge (44). Furthermore, innate immune responses are undoubtedly important in resistance against Mtb (45) and increasing evidence suggests a role for antibodies in protection (46–48). There is a fine balance between protective immune responses and excess immunopathology, and the type of immune responses induced is critical, particularly as Mtb can infect recruited myeloid cells such as neutrophils and myeloid-derived suppressor cells (49, 50). The immunology of Mtb infection is illustrated in simplified form in Figure 1. The strengths of BCG as a vaccine are that it induces immune responses against a broad range of mycobacterial antigens, boosts innate immunity by stimulating monocytes, persists for a relatively long time compared to non-live vaccines, and requires no adjuvants (51, 52). Its failure to provide sufficient protection against TB may be related to insufficient generation of CD8^+^ T-cell responses and CD4^+^ T_CM_ cell responses, which are required for long-term protection against Mtb (41, 53–57). It has been proposed that BCG fails to provide long-term protection, as the rate of TB increases in early adulthood and some studies have shown waning of protection after 10–15 years (25, 58). However, recent meta-analyses suggest that BCG can be effective for 20 years or longer in some populations (19). This is similar to, or even better than, the duration of protection of other commonly used vaccines, which often require boosters every 10 years. However, boosting BCG with repeat doses does not seem to be effective (59). In individuals infected with Mtb or constantly exposed to environmental mycobacteria, vaccine-induced T_CM_ cells are under increased pressure and constant exposure to antigens may deplete pools of T_CM_ cells by stimulating them to differentiate into effector cells (56, 58).

In both mice and humans, the T-cell responses to BCG vaccination are dominated by effector or T_EM_ cells rather than T_CM_ cells (57, 58). BCG resides in the phagosome of host cells, and its antigens are therefore primarily processed by major histocompatibility complex (MHC) class II pathways, stimulating CD4^+^ T-cell responses (60). BCG is also a poor inducer of apoptosis, a process of controlled cell death, which promotes the induction of both CD4^+^ and CD8^+^ T cells (53, 55, 61). In mice, the loss of BCG-mediated protection in the chronic phase coincides with a loss of CD4^+^ T_CM_ cells and an increase in terminally differentiated, dysfunctional T cells (41, 54). Mtb is a slow growing bacterium, and the chronic nature of the disease may lead to T-cell exhaustion—a progressive loss of T-cell function. Thus, an effective vaccine should induce large pools of memory cells that can replenish T_EM_ cells. In humans, increasing mycobacterial load coincides with progressive impairment of Mtb-specific CD4^+^ T-cell responses (62) (41, 54, 63, 64). As exhausted T cells can be restored by inhibiting programmed cell death protein (PD)-1 or stimulating toll-like receptor 2, it is possible that host-directed therapy to improve T-cell function could be used therapeutically in TB patients, but studies are still ongoing (65). Recently, it was shown that memory CD4^+^ T cells recruited to the lung attenuated Mtb growth in the early stages of disease, but their interaction with Mtb-infected macrophages did not promote their continued proliferation, resulting in only transient protection followed by waning immunity (66). Furthermore, Mtb-infected dendritic cells (DCs) cannot efficiently present antigens and instead transfer antigens to bystander DCs in the lymph nodes, which present the antigens to T cells (67–69). This causes a delay in activation of memory T cells and their recruitment to the lung. Overcoming such obstacles to sterilizing immunity against Mtb would improve the efficacy of TB vaccines markedly (68).

Novel vaccines aim to increase the number and quality of T_RM_ and T_CM_ cells generated (13, 70–72). While it was first thought that only live vaccines could generate good T_CM_ responses, novel adjuvants administered with Mtb antigens have also shown success in this regard (41, 70). In clinical trials of novel vaccine candidates, it will be important to monitor long-term protective efficacy in populations with different levels of exposure to Mtb (58).

## rBCGs Against TB

A number of rBCGs have been generated and tested for immunogenicity and/or efficacy in animal models. To narrow it down, we will discuss primarily those that have been tested for protective efficacy against Mtb (see Table 1). This includes BCG strains that have been engineered to induce increased immune responses by insertion of genes for Mtb antigens, mammalian cytokines or host resistance factors, insertion of bacterial toxin derived adjuvants, and manipulation of bacterial genes in order to increase antigen presentation and immune activation.

**Table 1:** Recombinant bacille Calmette-Guérin (rBCG) vaccine candidates and their protective efficacy against *Mycobacterium tuberculosis* (Mtb) challenge.

Name	Description	Results of testing	Reference
BCG:RD1-2F9/BCG:RD1/BCG:ESX-1	Bacille Calmette-Guérin (BCG) with the RD1 gene cluster inserted (Rv3861–Rv3885). Expresses early secretory antigenic target-6 (ESAT-6) and culture filtrate protein-10 (CFP10)	Mice: comparable efficacy in the lungs, moderate decrease in spleen bacterial burdens. Increased virulence Guinea pigs: strong decrease in spleen bacterial loads, reduced pathology	(73, 74)
rBCG E6	rBCG over-expressing antigen ESAT-6	Guinea pigs: comparable protection to BCG	(75)
rBCG30	rBCG over-expressing antigen 85B (Ag85B)	Guinea pigs: increased survival Safe and immunogenic in Phase I clinical trials	(76, 77)
rBCG(mbtB30)	rBCG over-expressing Ag85B, with disrupted synthesis of the siderophore mycobactin, preventing normal iron acquisition	Guinea pigs: slight decrease in bacterial burdens Mice: safer in severe combined immunodeficiency (SCID) mice	(78)
rBCG-1173:A	rBCG expressing Ag85A	Mice: slight decrease in bacterial burdens	(79)
rBCG:XB	rBCG expressing Ag85B and latency antigen HspX	Mice: strong decrease in bacterial burdens	(80)
(H)PE-ΔMPT64-BCG	rBCG expressing MPT64 fused to the PE domain of the PE_PGRS33 protein of Mtb that localizes to the cell wall	Mice: slight decrease in bacterial burdens	(81)
BCG:ESAT-L28A/L29S	rBCG:ESX-1 variant with mutations in the ESAT-6 gene	Mice: moderate decrease in spleen bacterial burdens, no difference in lung bacterial burdens. Attenuated Guinea pigs: strong decrease in spleen bacterial burdens	(82)
BCG:ESX-1^Mmar^	rBCG with the insertion of the *esx1* locus of *Mycobacterium marinum*	Mice: moderate reduction in bacterial loads in lungs and spleens after virulent Mtb challenge. As safe as BCG	(83)
BCG-IL-4, BCG-IL-6, BCG-GM-CSF, BCG-IFN-γ, BCG-IL-2	rBCG expressing murine interleukin (IL)-4, IL-6, GM-CSF, IFN-γ, and IL-2	No efficacy data	(84)
BCG secreting IL-2	BCG secreting IL-2	No efficacy data	(85)
rBCG-mIL-18	BCG secreting mouse IL-18	Mice: attenuated. Efficacy against Mtb not tested	(86)
BCG-IL-18	BCG secreting mouse IL-18	Mice: no difference in virulence compared to BCG. Efficacy against Mtb not tested	(87)
rBCG/IL-18	rBCG expressing IL-18	Mice: decreased protective efficacy against virulent *Mycobacterium bovis* challenge compared to BCG	(88)
rBCG/IL-2	rBCG expressing IL-2	Mice: did not increase protective efficacy against the virulent *M. bovis* challenge compared to BCG	(88)
BCG-IFN-gamma	rBCG secreting murine IFN-γ	Mice: did not increase protective efficacy against Mtb compared to BCG	(89)
rBCG-Ag85B-IL-15	rBCG expressing a fusion protein of Ag85B and IL-15	Mice: decreased bacterial burdens after intratracheal Mtb challenge, compared to BCG-Ag85B. No comparison performed with BCG	(90)
rBCG-Ag85B-ESAT-6-TNF-α	rBCG expressing the fusion protein Ag85B-ESAT-6-TNF-α	Efficacy against Mtb not tested	(91)
rBCG Ag85B-ESAT-6-IFN-γ	rBCG strain expressing the fusion protein Ag85B-ESAT-6-IFN-γ	Mice: slightly reduced bacterial burdens compared to BCG	(92)
BCGi	The *Ipr1* (intracellular pathogen resistance 1) gene was inserted into BCG	Mice: decreased bacterial loads in the lungs and spleens after Mtb challenge	(93)
rBCG(MCP-3)	Insertion of the gene for the chemokine monocyte chemotactic protein 3 (MCP-3) into BCG	Mice: increased safety in immunodeficient mice. Efficacy comparable to BCG	(94)
VPM1002 (BCG *ΔureC*:hly)	BCG expressing the *Listeria monocytogenes* protein listeriolysin O (LLO) instead of urease C	Mice: increased safety in both immunocompetent and immunodeficient mice. Moderate to strongly decreased bacterial loads and pathology compared to BCG Safe in guinea pigs, rabbits, and non-human primates Successfully passed Phase II clinical trials	(42, 95–97)
BCG *ΔureC*:*hly ΔnuoG*/VPM1002 *ΔnuoG*	BCG *ΔureC*:*hly* with deletion of *nuoG*, an anti-apoptotic gene	Mice: strong decrease in bacterial burdens compared to BCG, slight decrease in bacterial burdens compared to VPM1002 Safety equivalent to VPM1002	(96)
rBCGΔ*ureC*::*hly* (pMPIIB01)	BCG *ΔureC*:*hly* expressing the latency antigens Rv2659c, Rv3407, and Rv1733c	Mice: moderately reduced bacterial burdens compared to the parental strain in both lung and spleen; decreased lung pathology. Comparison to BCG not performed	(98)
BCG Δ*ureC*:*hly Δpdx1*/VPM1002 *Δpdx1*	BCG Δ*ureC*:*hly* deficient in pyridoxine synthase, an enzyme required for synthesis of the essential vitamin B6	Mice: safer in immunocompromised and wild-type mice compared to the parental strain. Homologous prime–boost regimen afforded similar protection to BCG	(99)
BCG Δ*ureC*:*hly_*hIL7 and BCG Δ*ureC*:*hly*_hIL18	BCG Δ*ureC*:*hly* expressing human IL-7 or human IL-18	Mice: no increase in efficacy compared to the parental strain	(100)
AERAS-401 (BCG1331 Δ*ureC*:Ω*pfoAG137Q)*	BCG with the *ureC* gene replaced with the *PfoAG137Q* gene for perfringolysin O	Mice: safe in immunocompromised SCID mice. No increase in efficacy compared to BCG	(101)
AERAS-422 (AFRO-1)	AERAS-401 incorporating genes coding for Ag85A, Ag85B, and TB10.4	Mice: challenge with a virulent Mtb strain demonstrated increased survival after immunization with AERAS-442 compared to BCG Clinical trials discontinued [associated with the development of shingles (varicella-zoster virus reactivation)]	(101)
rBCG-LTAK63lo	rBCG expressing LTAK63, a detoxified form of *Escherichia coli* heat labile toxin	Mice: greatly reduced bacterial burdens compared to BCG. At a high challenge dose, mice immunized with rBCG-LTAK63lo had reduced bacterial loads and increased survival. rBCG-LTAK63lo also increased protection against challenge with a virulent Mtb Beijing isolate	(102)
BCG Δ*zmp1*	BCG deficient in gene *zmp1*, a putative Zn(2+) metalloprotease	Guinea pigs: slight reduction in lung bacterial loads compared to BCG	(103)

*The table summarizes results from the testing of rBCG against TB in animal models. Unless otherwise specified, Mtb challenge was performed. Decreases in bacterial burdens were estimated from graphs if not specified and listed as follows for comparative purposes: up to 0.5 log decrease: slight; 0.5 to 1.0 log decrease: moderate; over 1.0 log decrease: strong*.

### rBCGs Expressing Mtb Antigens

Analysis of the genetic differences between BCG and Mtb has determined that 16 genomic regions of difference (designated RD1–RD16) have been deleted from BCG substrains, although some substrains do contain RD2, RD8, RD14, and RD16 (104–106). RD1 to RD3 were the first to be identified and are present in virulent *M. bovis* as well as Mtb (104). RD1 is a 9.5-kb segment deleted from all BCG substrains but conserved in all virulent isolates of *M. bovis* and Mtb, and it regulates multiple genetic loci. The RD1 segment contains the genes for the immunodominant antigens early secretory antigenic target (ESAT)-6 and culture filtrate protein-10 (CFP-10), as well as components of the type VII ESAT-6 secretion system (ESX)-1 required to secrete them (73, 104, 107, 108). RD1 deletion and the loss of the ESX-1 secretion system was a major factor in the original attenuation of BCG (73, 104, 107). At least five additional T-cell antigens are encoded by RD1 (PE35, PPE68, Rv3871, Rv3878, and Rv3879c), suggesting that RD1 constitutes an immunogenicity island (108). RD2 is a 10.7-kb segment deleted only from substrains derived from the original BCG Pasteur strain after 1925, and it is conserved in *M. bovis* and Mtb. RD2 contains novel repetitive elements and the *mpt64* gene, encoding the protein MPT64 which elicits T-cell responses and delayed hypersensitivity reactions in Mtb-infected patients (109). Finally, RD3 is a 9.3-kb segment absent from BCG, present in virulent laboratory strains of *M. bovis* and Mtb, but absent from 84% of virulent clinical isolates. The loss of RD1 to RD16 means that BCG lacks a number of the antigens of Mtb, and attempts have been made to improve the efficacy of BCG against TB by generating rBCGs expressing antigens particular to Mtb, such as ESAT-6 (74, 75, 80, 81, 110). ESAT-6 not only acts as an antigen but also can induce IL-18-dependent IFN-gamma secretion by Mtb antigen-independent memory CD8^+^ T cells and NK cells (111). Furthermore, rBCGs over-expressing antigens that are found in both BCG and Mtb, such as antigen 85B (Ag85B), have been generated in an attempt to boost immune responses against shared mycobacterial antigens. In support of the fact that including Mtb antigens improves the protective efficacy of mycobacterial strains, removing CFP-10 and ESAT-6 from the attenuated Mtb-derived MTBVAC strain reduced its protection against Mtb in mice to that of BCG levels (112). MTBVAC is an Mtb strain attenuated by independent deletions of the *phoP* and *fadD26* virulence genes.

An rBCG over-expressing ESAT-6 induced stronger IFN-γ responses than parental BCG but did not improve protection against aerosol Mtb challenge in guinea pigs (75). In contrast, rBCG30, over-expressing the shared immunodominant Ag85B, improved protection against Mtb in a guinea pig model relative to BCG (76). Ag85B is one of the three similar secreted mycolyl-transferases that are important for bacterial wall synthesis (113). A Phase I clinical trial was completed where 35 adults were randomized to receive rBCG30 or parental BCG in a double-blind fashion (77). The vaccine was well tolerated, and expansion and IFN-gamma production of Ag85B-specific CD4^+^ and CD8^+^ T cells were increased in the rBCG30 immunized individuals compared to those immunized with parental BCG (77). To improve the safety of rBCG30 for use in immunocompromised individuals, a new construct, rBCG(mbtB30) was developed, which has disrupted synthesis of the siderophore mycobactin, preventing normal iron acquisition (78). This strain is mycobactin-dependent but can undergo limited replication if sufficient ferric mycobactin is provided. It was shown to be safer than BCG in immunocompromised severe combined immunodeficiency (SCID) mice and was slightly more protective against Mtb than parental BCG in guinea pigs (78).

Other rBCGs expressing Mtb antigens that provided increased protection against Mtb in mice include rBCG expressing Ag85A (79), rBCG expressing Ag85B and latency antigen HspX (rBCG:XB)(80), and rBCG expressing MPT64 fused to the PE domain of the PE_PGRS33 protein of Mtb that localizes to the cell wall ((H)PE-ΔMPT64-BCG) (81). In another approach, co-administration of an rMTb72F fusion protein (composed of Mtb32 and Mtb39 antigens) in an AS02A adjuvant together with BCG increased survival and decreased lung pathology in guinea pigs (110). A small number of antigens feature frequently in the makeup of vaccine candidates generated for pre-clinical and clinical evaluation, with six of the eight subunit vaccines in clinical trials containing an Ag85 protein (14). Current research therefore aims to identify novel antigens and broaden antigen selection for vaccine design.

### rBCGs Complemented with ESX-1 Variants

As mentioned in the previous section, BCG lacks the RD1 and thus a functional ESX-1 secretion system and the antigens ESAT-6 and CFP-10, which form a heterodimeric complex and act as virulence factors (73, 104, 107, 108). ESX-1 is involved in host–pathogen interactions, as access of bacterial antigens to the cytosol influences both bacterial virulence and host immune recognition (73, 83, 114). An rBCG complemented with the complete RD1 locus (BCG:RD1, also known as BCG:ESX-1) provided better protection against Mtb challenge in both mice and guinea pigs than parental BCG, with reduced lung pathology and dissemination of bacteria (74). However, insertion of the Mtb *esx-1* locus into BCG leads to increased virulence in immunodeficient mice and prolonged persistence in immunocompetent mice (74). This issue was approached by introducing mutations into the *esxA* gene (encoding ESAT-6) of BCG:ESX-1 (82) or by inserting the *esx-1* locus of *Mycobacterium marinum*, which has reduced virulence, into BCG (83). A BCG:ESAT-L28A/L29S strain carrying modifications at residues Leu(28)-Leu(29) of the ESAT protein was strongly attenuated in mice and demonstrated protective efficacy against Mtb challenge in mice and guinea pigs, with similar lung bacterial burdens but a strong reduction in spleen bacterial burdens (82). BCG:ESX-1^Mmar^ increased protective efficacy compared to BCG in mice, demonstrating similar immunogenicity and efficacy to BCG:ESX^Mtb^ but was as safe as parental BCG (83). Compared to BCG Danish, BCG:ESX-1^Mmar^ reduced bacterial loads in lungs and spleens by an additional log after a challenge with Mtb HN878 (Beijing family) and Mtb strain M2 (Harlem family). Expression of ESX-1^Mmar^ induced the cGas/STING/TBK1/IRF-3/type I interferon axis and enhanced AIM2 and NLRP3 inflammasome activity, leading to increased proportions of mycobacteria-specific cytokine-producing CD4^+^ Th1 cells and CD8^+^ T cells.

### rBCGs Expressing Host Immunomodulatory Molecules

In an effort to increase host responses to mycobacterial antigens following BCG vaccination, a number of rBCGs have been generated expressing functional mammalian cytokines or other host molecules (84, 85, 87, 93). Cytokine-producing rBCG strains have also been generated with the aim of testing them in intravesical BCG immunotherapy for bladder cancer, which seems to require the activation of Th1 responses for efficacy (86, 115, 116). Most murine cytokines tested could be produced and secreted by BCG and were functional (84). Splenocytes stimulated with IL-2 secreting BCG produced increased IFN-γ compared to those stimulated with BCG (85). A study tested rBCGs expressing murine IL-4, IL-6, GM-CSF, IFN-γ, and IL-2 and found that BCGs secreting IL-2, IFN-γ, or GM-CSF were more potent stimulators of responses to PPD than BCG, with splenocytes from mice immunized with these strains producing large amounts of IFN-γ (84). In addition, two independent studies demonstrated that rBCG strains expressing IL-18, a cytokine that acts in synergy with IL-12 to induce IFN-γ (88), promoted IFN-γ production by splenocytes in response to PPD (86, 87). In one of these studies, mice infected with rBCG-mIL-18 had decreased bacterial loads compared to mice infected with parental BCG (86), whereas in the other, they did not (87). None of the aforementioned strains were tested for protective efficacy against Mtb challenge; however, rBCGs expressing IL-2 or IL-18 were tested against infection with virulent *M. bovis* (88). In this study, rBCG/IL-18 induced less IFN-γ than BCG and had lower protective efficacy against *M. bovis* challenge compared to BCG. Despite inducing increased Th1 responses and protecting against intranasal challenge with BCG, immunization with rBCG/IL-2 did not increase protective efficacy against the virulent *M. bovis* challenge. Similarly, when IFN-γ-deficient mice were infected via aerosol with rBCG secreting murine IFN-γ, they had reduced bacterial loads and more differentiated granulomas compared to mice infected with the control rBCG containing vector only, demonstrating the potential to influence disease outcomes (89). However, subsequent challenge of IFN-γ^−/−^ mice with Mtb demonstrated that rBCG secreting IFN-γ did not provide additional protection against Mtb compared to BCG-vector. In this study, IFN-γ was only produced locally and local production was insufficient for improving systemic immune responses. Overall, conferring upon BCG, the ability to secrete cytokines does not seem to enhance its protective efficacy, probably because of the amount of cytokine secreted and its locality.

In another strategy, rBCGs expressing combinations of cytokines and antigens, sometimes in the form of fusion proteins (90–92), have also been generated. The rBCG strain expressing the fusion protein Ag85B-ESAT-6-IFN-γ only slightly reduced bacterial burdens after Mtb challenge compared to BCG (92). rBCG-Ag85B-ESAT-6-TNF-α increased IFN-γ secreting cells, but protection was not measured (91). More promising was an rBCG expressing a fusion protein of Ag85B and IL-15, a cytokine important for the proliferation and survival of memory CD8^+^ T cells (rBCG-Ag85B-IL-15), which was found to increase numbers of IFN-γ-producing CD44^+^CD4^+^ and CD44^+^CD8^+^ T cells and decrease bacterial burdens after intratracheal Mtb challenge, compared to BCG-Ag85B, although comparison to BCG was not performed (90).

Genes for other host-derived immunoregulatory molecules have also been inserted into BCG. In one study, the *Ipr1* (intracellular pathogen resistance 1) gene was inserted into BCG to produce rBCGi (93). *Irp1* is an IFN-regulated gene that enhances macrophage resistance to intracellular pathogens such as Mtb and *Listeria monocytogenes* and is not expressed in susceptible C3HeB/FeJ mice (117). Vaccination of C3HeB/FeJ mice with rBCGi was more protective against Mtb infection than BCG, with decreased bacterial loads in the lungs and spleens of BCGi-vaccinated mice. Gene expression analysis of 113 immune-related genes demonstrated 20 differentially expressed genes with greater than twofold change between rBCGi and BCG-vaccinated groups. In another study, insertion of the gene for the chemokine monocyte chemotactic protein 3 (MCP-3) into BCG did not improve its efficacy, but increased its safety, since immunodeficient mice infected with rBCG(MCP-3) survived longer than mice infected with the parental BCG (94).

### VPM1002 and Second-Generation Derivatives

**Figure 2:**
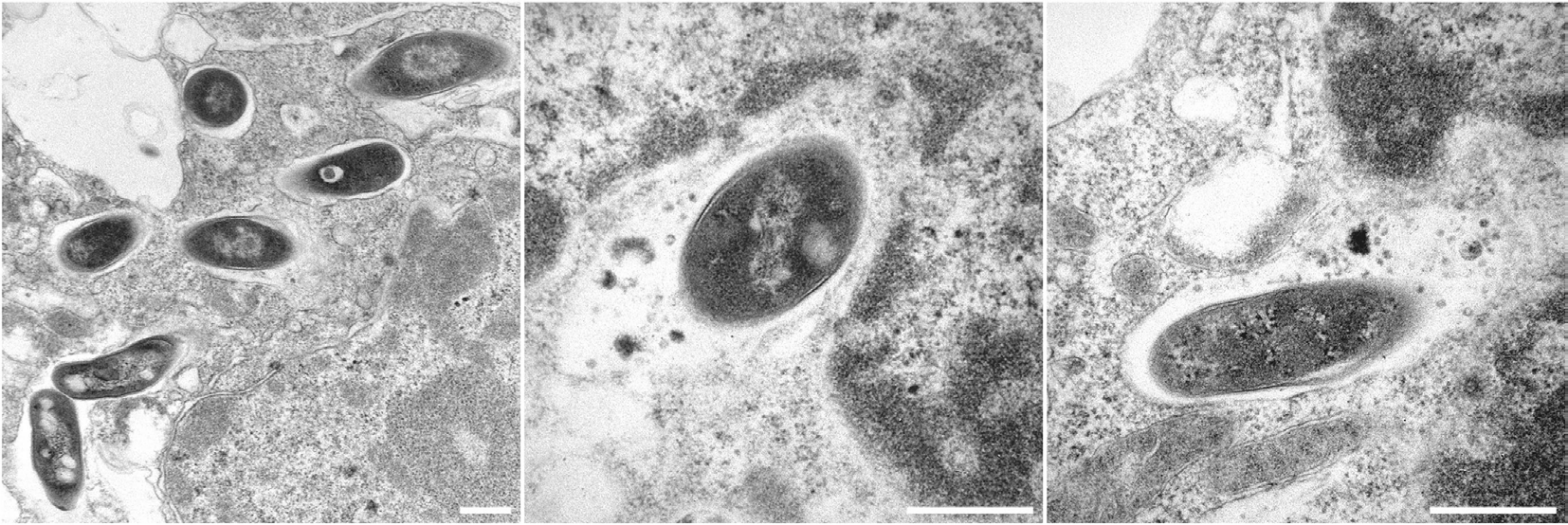
Electron micrographs showing Bacille Calmette-Guérin (BCG) *ΔureC:hly* inside host macrophages. All scale bars represent 0.5 µm. Left panel: BCG *ΔureC:hly* within phagosomes after uptake by host cells. Middle and right panels: Phagolysosomal fusion after the infection of host cells with recombinant BCG *ΔureC:hly*.

In previous reviews, we have discussed the development of VPM1002 (BCG *ΔureC*:*hly*) from the laboratory through to clinical trials in detail (118, 119). Essentially, VPM1002 is an rBCG that has been engineered to express the *L. monocytogenes* protein listeriolysin O (LLO) instead of urease C. Urease C inhibits acidification of the phagosome by converting urea to ammonia, preventing phagosome maturation (120–122). This activity inhibits trafficking of MHCII to the cell surface resulting in a reduced MHCII expression and antigen presentation (120). LLO is a cholesterol-binding, pore-forming protein that allows escape of *L. monocytogenes* from the phagosome (123). It requires acidic pH for optimal activity, which can be achieved by deletion of urease C (95). Expression of LLO in the rBCG causes perturbation to the phagosome (Figure 2) and leakage of bacterial DNA into the cytosol, triggering activation of the AIM2 inflammasome, and increased autophagy and apoptosis (124). Access of bacterial antigens to the cytosol increases its availability to the MHC class I antigen presentation pathway and promotes cross-presentation (69, 125). In mice, VPM1002 induces better protection, providing a strong increase in efficacy compared to BCG (42, 96). It also provides protection as a post-exposure vaccine (97). Furthermore, it is attenuated, with increased safety in both immunocompetent and immunodeficient mice (95, 96). Phase I and Phase II clinical trials have demonstrated its safety and immunogenicity in humans, including neonates, and a Phase II/III efficacy trial as a vaccine against recurrent TB has commenced (119).

While VPM1002 has progressed through the development pipeline, next-generation derivatives of this promising candidate have been developed and tested in pre-clinical studies, with the aim of further enhancing immunogenicity and/or safety (126). The most successful of these so far is VPM1002 *ΔnuoG*, which increased protection compared to its parental strain rBCG *ΔureC*:*hly* in mice while maintaining safety (96). The *nuoG* gene codes for a subunit of the non-essential respiratory enzyme complex NADH dehydrogenase type I and was identified as an anti-apoptotic virulence gene after gain-of-function screening using an *M. smegmatis* mutant library following by generation of Mtb H37Rv mutants (61). Deletion of *nuoG* from Mtb decreased its virulence and lead to increased apoptosis at day 14 post-infection. The mechanism was TNF-α dependent and relied on NADPH oxidase (NOX2)-dependent reactive oxygen species (ROS) (127). Subsequently, it was found that deletion of the *nuoG* gene from both BCG and VPM1002 enhanced protective efficacy against Mtb in mice, with VPM1002 *ΔnuoG* having strongly increased efficacy compared to BCG-vaccinated mice (96). Intriguingly, as well as increasing apoptosis, deletion of *nuoG* also increased the recruitment of the autophagy protein microtubule-associated protein 1A/1B-light chain 3 (LC3) to the live vaccine strains, suggesting that type I NADH dehydrogenase has an additional role in inhibition of autophagic pathways. LC3-associated phagocytosis, which involves conjugation of LC3 to phagosomes and subsequent phagolysosomal fusion, requires NOX2 activity and the production of ROS. Therefore, as type I NADH dehydrogenase inhibits ROS, it is possible that it also inhibits LC3-associated phagocytosis. However, similar persistence of VPM1002 and VPM1002 *ΔnuoG* strains in mice suggested that inflammasome or apoptosis-mediated mechanisms induced by both strains were primarily responsible for the strain attenuation. The increased efficacy of VPM1002 *ΔnuoG* compared to VPM1002 was associated with increased CD4^+^ T_EM_, follicular T helper cells and germinal center B cells (96).

Another approach to improve the efficacy of VPM1002 was to express the latency antigens Rv2659c, Rv3407, and Rv1733c in this strain (98). Rv2659c, encoded by Mtb and not BCG, is expressed during nutrient deprivation, while Rv1733 and Rv3407, present in both BCG and Mtb, are expressed during hypoxia and reactivating disease in a mouse model of TB, respectively. Expression of the three latency antigens in the rBCGΔ*ureC*∷*hly* (pMPIIB01) strain improved long-term efficacy against challenge with a clinical Mtb strain, with reduced bacterial burdens compared to the parental strain in both lung and spleen at day 200 post-infection after intra-dermal vaccination of mice, as well as decreased lung pathology. The strain was not compared to BCG but showed a strong reduction in bacterial loads compared to unvaccinated mice.

Another second-generation construct, BCG *ΔureC*:*hly Δpdx1*, or VPM1002 *Δpdx1*, was generated in an attempt to further improve the safety of the vaccine so that it might be suitable for immunization of immunocompromised individuals such as HIV^+^ infants and adults and HIV-exposed neonates, who are at higher risk of developing TB (99). VPM1002 *Δpdx1* is deficient in pyridoxine synthase, an enzyme required for synthesis of the essential vitamin B6, and therefore is auxotrophic for vitamin B6 in a concentration dependent manner. VPM1002 *Δpdx1* was profoundly attenuated, being safer in immunocompromised SCID mice compared to its parental strain. In addition, it demonstrated reduced dissemination in wild-type mice, which was partially reversed by dietary supplementation with vitamin B6. Immune responses to the strain were also dependent on vitamin B6 supplementation. Protective efficacy against Mtb was similar to BCG at day 30 but was lost by day 180; however, a homologous prime–boost regimen afforded similar protection to BCG. Protection depended on a dietary source of vitamin B6 at early time points following Mtb challenge, but protection at 180 days post-challenge with Mtb H37Rv and 160 days post challenge with Mtb Beijing/W remained independent from vitamin B6 supplementation, suggesting that it relied on immune responses generated at early time points.

As discussed earlier, there are several examples of rBCGs expressing mammalian cytokines, which promote increased immune responses (116). Therefore, Rao et al. attempted to further increase the immunogenicity of BCG *ΔureC*:*hly* by generating BCG *ΔureC*:*hly* derivatives expressing the cytokines IL-7 and IL-18 (100). IL-7 and IL-18 play a role in immunity to Mtb infection (128–130). IL-7 is required for T-cell development (131), and rIL-7 influences recall T-cell responses to Mtb infection (132). IL-18 induces Th1 responses (including IFN-gamma and TNF-α secretion), together with IL-12 (133). IL-18-deficient mice are susceptible to TB (128, 134) and BCG infection (134). Previously, splenocytes of mice vaccinated with rBCG expressing IL-18 produced higher amounts of Th1 cytokines after stimulation with mycobacterial antigens than splenocytes of mice vaccinated with parental BCG (87). Furthermore, increased IL-18 mRNA was detected after vaccination with BCG *ΔureC*:*hly* and BCG *ΔureC*:*hly ΔnuoG*, which have increased efficacy compared to BCG (96, 124).

Growth assays demonstrated that expression of IL-7 or IL-18 did not compromise intracellular fitness of BCG Δ*ureC*:*hly_*hIL7 and BCG Δ*ureC*:*hly*_hIL18 (100). Secretion of pro-inflammatory cytokines including IL-6, TNF-α, and G-CSF was increased in DCs infected with BCG Δ*ureC*:*hly*_hIL18 compared to BCG, although in co-cultures T-cell activation was not influenced. Similarly, BCG Δ*ureC:hly*_hIL18 immunized mice showed up-regulation of pro-inflammatory cytokines IL-6, KC, CCL5, IL-2, and G-CSF compared to those vaccinated with BCG. At day 60, all strains on the BCG *ΔureC*:*hly* background induced similar numbers of CD40L-expressing CD4^+^ T cells in the lungs, but BCG Δ*ureC*:*hly*_hIL18 and BCG Δ*ureC*:*hly*_hIL7 induced increased CD40L^+^TNF-α^+^ and CD40L^+^TNF-α^+^IFN-γ^+^ CD4^+^ T cells compared to BCG Δ*ureC*:*hly* between 30 and 60 days post-vaccination. Efficacy, measured by bacterial loads, was comparable to the parental strain. Therefore, expression of hIL-7 or hIL-18 by VPM1002 did not further improve protection. Suggested reasons were poor secretion of hIL-7 and hIL-18 and overload of the mycobacterial export machinery due to the use of the same export system (PgroEL2-Ag85BSS) for both LLO and the cytokines.

VMP1002, VPM1002 *ΔnuoG*, and VPM1002 *Δpdx1* are currently being tested for safety and protective efficacy against *M. caprae* infection in goats by the Friedrich Loeffler Institute in Germany (Menge et al., unpublished data). Results should be available in 2018.

## AEREAS-401 and AERAS-422

AERAS-401 is an rBCG expressing the cholesterol-binding cytoslysin perfringolysin O (Pfo), a pore-forming protein normally secreted by *Clostrididium perfringens* (101, 135). Pfo interacts with membranes at both low and neutral pH, although low pH enhances Pfo membrane binding, oligomerization, and pore formation (136). Generation of this BCG strain (BCG1331 Δ*ureC*:Ω*pfoAG137Q*) was accomplished by replacing the *ureC* gene with the *PfoAG137Q* gene under the control of the Ag85B promoter (101). AERAS-401 secreted biologically active Pfo, associated with lysis of the endosome compartment, and had a good safety profile in immunocompromised SCID mice. A second-generation derivative of AEREAS-401, AREAS-422 (research strain AFRO-1), was then generated by incorporating genes coding for Ag85A, Ag85B, and TB10.4, into AERAS-401 in order to combine increased access of antigens to the cytosol with over-expression of Mtb antigens (101). AERAS-422 enhanced immune responses in both mice and guinea pigs compared to BCG. A short-term challenge experiment with a laboratory strain of Mtb in mice revealed no differences in bacterial loads in lungs and spleen after immunization with AERAS-422 compared with BCG, but challenge of vaccinated mice with the hypovirulent Mtb strain HN878 demonstrated increased survival after immunization with AERAS-442 compared to BCG (101). AERAS-422 was subsequently tested in Phase I clinical trials (137). It induced more potent immune responses than BCG, but immunization with AERAS-422 at the highest inoculum was associated with the development of shingles (varicella-zoster virus reactivation) in two of the eight healthy volunteers, and the study was discontinued. Whole blood stimulation with BCG demonstrated that both of the volunteers who developed shingles displayed five- to tenfold higher IFN-γ responses compared to the other recipients, and it was suggested that the effects of IFN-γ on type I IFN responses (required for antiviral immunity) should be investigated. In the trial, earlier and more robust NK and cytotoxic T-cell responses correlated with increased mycobacterial growth inhibition, suggesting that NK cells and cytotoxic T cells may serve as a target for improved vaccines against TB. In contrast, increased expression of myeloid and pro-inflammatory genes was negatively associated with mycobacterial growth inhibition.

### rBCGs Expressing Bacterial Toxins

Bacterial toxins and toxin derivatives possess immunostimulatory properties and activate immune responses to bystander antigens when present simultaneously, but their toxicity renders them unsuitable for use as adjuvants in humans (138). An example is cholera toxin (CT), which is a potent mucosal adjuvant but is not suitable for use in humans as its inflammatory nature induces adverse events (68). Mice immunized with rBCG expressing CT B developed increased levels of anti-BCG IgA and IgG responses compared to those immunized with parental BCG, associated with increased TGF-β production (139). In another study, CT enhanced IL-17 when administered together with BCG, and this was associated with increased protection against Mtb challenge (68). While CT cannot be used in humans, the study highlighted the potential role of mucosal adjuvants in protection against TB.

*Escherichia coli* heat labile toxin (LT) is another mucosal adjuvant, shown to promote antigen presentation, T-cell proliferation, cytokine production, and mucosal IgA and IgG responses (140). Detoxification of the A subunit by genetic modification results in a potent, non-toxic mucosal adjuvant with no toxicity in mice, guinea pigs, and macaques (138, 141–144). Detoxified LT has a good clinical safety record after oral and percutaneous administration, but nasal administration is not recommended because it is associated with an increased risk of transient peripheral facial nerve palsy (145, 146). Additional safety studies for other routes of administration would be prudent, considering the adverse effects after intranasal immunization were not detected in initial trials (144).

Recently, an rBCG (rBCG-LTAK63lo) was generated to express low levels of a non-toxic derivative of LT (LTAK63) (102). Vaccination with rBCG-LTAK63lo induced increased Th1 cytokines and IL-17 in the lung compared to BCG. After intratracheal challenge with a laboratory strain of Mtb, mice had greatly reduced bacterial burdens compared to BCG at day 30 post-challenge, and at a high challenge dose, mice immunized with rBCG-LTAK63lo had reduced bacterial loads and increased survival. rBCG-LTAK63lo also increased protection against challenge with a virulent Mtb Beijing isolate.

### BCG Δ*zmp1*

Interleukin-1β is a major pro-inflammatory cytokine that is activated by cleavage of a pro-IL-1β precursor by caspases activated by assembly of the inflammasome, an inflammatory caspase-activating multi-protein complex (147). Mtb inhibits inflammasome activation and IL-1β processing by a mechanism involving the product of the virulence gene *zmp1*, a putative Zn(2+) metalloprotease (147). Accordingly, infection with Mtb deficient in *zmp1* triggers activation of the inflammasome, increased IL-1β secretion, enhanced maturation of phagosomes, and improved mycobacterial clearance by macrophages. Furthermore, *zmp1*-deficient Mtb was attenuated compared to the *zmp1^+^* parental strain, showing reduced bacterial burdens in the lungs after aerosol challenge. A BCG mutant strain deficient in *zmp1* also showed increased phagosome maturation and phagolysosome fusion (147, 148). This was demonstrated to facilitate antigen presentation and to increase mycobacteria-specific CD4^+^ and CD8^+^ T-cell responses, emphasizing that phagolysosome fusion is important for generating immune responses to BCG antigens (148). Two *zmp1*-deficient strains, BCG Pasteur Δ*zmp1*::*aph* and BCG Danish Δ*zmp1*, induced increased IFN-γ^+^CD4^+^ T-cell responses in cattle compared to BCG (149). Efficacy testing in guinea pigs demonstrated that *zmp1*-deficient BCG strains were more protective than BCG, with BCG Pasteur SmR *zmp1*::*aph* and BCG Denmark Δ*zmp1*, further reducing lung bacterial loads compared to their parental BCG strains (103). Furthermore, the *Δzmp1* mutants showed increased safety in immunocompromised SCID mice compared to BCG.

## Boosting BCG with Subunit Vaccines

Originally, it was thought that subunit protein vaccines had the potential to replace BCG, but evaluation of an extensive number of subunit vaccine candidates in animal models has demonstrated that at best, these match the protection afforded by BCG (150–152). In general, a survey of the literature shows that protection afforded by subunit vaccines is not as effective as that induced by live attenuated mycobacterial strains. However, subunit vaccines can increase protective efficacy when administered as boosters to a BCG prime and, thus, have an important role to play in TB vaccination strategies. Furthermore, testing does not necessitate withholding the BCG vaccine, which has been shown to be partially effective. Most people are vaccinated against BCG in infancy, and protection wanes after approximately 20 years (19). Table 2 summarizes the results of preclinical efficacy testing of some of the subunit vaccines that have been tested as BCG boosters. In some cases, subunit vaccine boosters have been used with an rBCG prime. This is by no means an exhaustive list but serves to illustrate the type of subunit vaccines being tested. The majority of the subunit vaccine boosters that reduce bacterial burdens compared to BCG in mice or guinea pigs only reduce them slightly, but some have also shown beneficial effects on survival and lung pathology. The ability to induce high levels of cytokine-producing CD4^+^ and CD8^+^ T cells does not necessarily correlate with protection, as in some cases vaccine candidates were very immunogenic but did not reduce bacterial loads or pathology (75, 153).

**Table 2:** Boosters to bacille Calmette-Guérin (BCG).

Prime	Boost	Protective efficacy versus BCG alone	Reference
BCG	Nanoemulsion mucosal adjuvant with early secretory antigenic target-6 (ESAT-6) and antigen 85B (Ag85B)	Mice: bacterial loads similar, reduced lung pathology	(154)
BCG	H56 fusion protein (Ag85B-ESAT-6-Rv2660c) with CAF01 adjuvant	Mice: moderate decrease in bacterial burdens	(155)
BCG	H1 fusion protein (Ag85B-ESAT-6) with CAF01 adjuvant	Mice: moderate to strong decrease in bacterial burdens	(41, 155)
BCG	H4 fusion protein (Ag85B-TB10.4) with IC31 adjuvant	Guinea pigs: moderate decrease in bacterial burdens, increased survival Mice: decreased bacterial burdens (slight in lungs, moderate in spleens)	(156, 157)
BCG	H56 fusion protein (Ag85B-ESAT-6-Rv2660c) with IC31 adjuvant	Macaques: reduced lung pathology, clinical disease and extrapulmonary dissemination, increased survival, prevention of reactivation of latent infection	(158)
BCG	Ag85B-ESAT-6 with LTK63 adjuvant	Mice: slight decrease in bacterial burdens	(141)
BCG	Mtb72F fusion protein DNA (Mtb32 and Mtb39 antigens)	Guinea pigs: decreased lung pathology	(110)
BCG	ID93 fusion protein (Rv1813, Rv2608, Rv3619, and Rv3620) with glucopyranosyl lipid adjuvant (GLA) stable emulsion (SE)	Guinea pigs: reduced pathology, increased survival	(159)
BCG	*Mycobacterium tuberculosis* (Mtb) antigens Rv0447, Rv2957, and Rv2958 (no adjuvant)	Mice: slight decrease in lung bacterial burdens	(16)
BCG	CMFO fusion protein (Rv2875-Rv3044-Rv2073c-Rv0577) with DMT adjuvant	Mice: strong decrease in bacterial burdens, protection after reactivation by glucocorticosteroids	(160)
BCG	SRL172/DAR-901 [inactivated whole cell booster from NTM (*Mycobacterium obuense*)]	Mice: no difference in bacterial loads	(161)
BCG	Rv2299c-ESAT-6 fusion protein	Mice: slight decrease in lung bacterial burdens, reduced pathology (HN878 challenge)	(151)
BCG	Human adenovirus 5 with Ag85A (Ad5Ag85A), i.m and i.n.	Mice: i.m. boosting did not increase protection, but intranasal boosting reduced lung and spleen bacterial burdens moderately and strongly, respectively	(162)
BCG	attenuated *Listeria monocytogenes* vector expressing Ag85B (rLm30)	Guinea pigs: no difference Mice: slightly reduced lung bacterial burdens	(163)
BCG	recombinant adenovirus vaccine expressing Ag85B (rAdv30)	Guinea pigs: no difference Mice: slightly reduced lung bacterial loads at early time point (6 weeks post-challenge) only	(163)
BCG	Six fusion proteins [ESAT-6-Ag85B-MPT64190-198-Mtb8.4 (EAMM), Ag85B-MPT64190-198-Mtb8.4 (AMM), Mtb10.4-HspX (MH), ESAT-6-Mtb8.4 (EM), Mtb10.4-Ag85B, ESAT-6-Ag85B (MAE), and ESAT-6-RpfE (ER)] in adjuvant composed of *N*,*N*′-dimethyl-*N*,*N*′-dioctadecylammonium bromide (DDA), polyribocytidylic acid (poly I:C), and gelatin	Mice: decreased lung pathology in EAMM boosted mice. Slightly decreased lung bacterial burdens in EAMM or AMM boosted mice	(164)
BCG	DNA encoding β-defensin 2 and antigens ESAT-6 and Ag8B (pDE and pDA)	Mice: increased survival, decreased lung pathology	(165)
BCG	Sendai virus with Ag85A and Ag85B	Mice: slightly reduced bacterial loads, reduced lung pathology	(166)
BCG	*Mycobacterium indicus pranii* (MIP), s.c. or aerosol	Guinea pigs: decreased lung pathology Mice: MIP boosters moderately reduced lung bacterial burdens	(167)
BCG	MVA85A (vaccinia based vector expressing Ag85A)	Mice: no difference Guinea pigs: no difference	(152, 168)
BCG i.n.	MVA85A i.n. or BCG i.n.	Mice: strong decrease in bacterial burdens	(169)
BCG	Chimpanzee adenovirus with Ag85A (ChAdOx185A), with and without MVA85A boost	Mice: lung bacterial burdens were slightly improved after intranasal boosting with both ChAdOx185A and MVA85A	(153)
rBCG expressing PPE protein Rv3425	Rv3425	Mice: slightly reduced lung bacterial burden, decreased lung pathology	(170)
rBCG secreting Ag85B-ESAT-6	LTK63-adjuvanted Ag85B-ESAT-6	Guinea pigs: no increase in protection	(142)
VPM1002	MVA85A	Mice: no difference	(168)
rBCG expressing ESAT-6	DNAE6 (ESAT-6 DNA)	Mice: reduced protection compared to prime alone	(75)

*The table illustrates some of the BCG boosters that have been tested against tuberculosis (TB) in animal models. Decreases in bacterial burdens were estimated from graphs if not specified and listed as follows for comparative purposes: up to 0.5 log decrease: slight; 0.5 to 1.0 log decrease: moderate; over 1.0 log decrease: strong. i.m., intramuscular; i.n., intranasal; s.c., subcutaneous*.

## Conclusion

Bacille Calmette-Guérin can contribute to the control of TB, being most effective in children and those not previously infected with Mtb or sensitized to environmental mycobacteria (17–19, 22). However, BCG fails to adequately protect those in high-risk environments, where the prevalence of Mtb is high and socio-economic conditions are poor. A wide variety of rBCG strains and subunit vaccines have been tested in pre-clinical trials. Only one rBCG is now in clinical trials (VPM1002), while other vaccine candidates in clinical trials include inactivated whole cell vaccines, attenuated mycobacterial strains and subunit vaccine boosters focusing mostly on a narrow range of antigens (Ag85 family, ESAT-6) (14, 15). Head-to-head testing of vaccine candidates in pre-clinical models may be useful for identifying the most promising candidates worth moving forward to clinical trials (152). A number of studies have revealed that the immunogenicity parameters measured often do not translate to increased efficacy, and hence pre-clinical trials should always include Mtb challenge (75, 153). Statistical analysis should be performed comparing novel candidates against BCG, the current clinical vaccine with proven partial efficacy in humans. In summary, it seems likely that an improved vaccination regimen against TB can be achieved, but overcoming the current limits in protective efficacy will require novel approaches.

## Author Contributions

Both authors wrote the manuscript and approved it for publication.

## Conflict of Interest Statement

SK is co-inventor/patent holder of VPM1002. NN has no conflicts of interest.

## Abbreviations

Ag85B, antigen 85B; BCG, *Mycobacterium bovis* bacille Calmette–Guérin; CFP-10, culture filtrate protein-10; CT, cholera toxin; DCs, dendritic cells; ESAT-6, early secretory antigenic target-6; ESX-1, ESAT-6 secretion system 1; IL, interleukin; LC3, microtubule-associated protein light chain 3; LLO, listeriolysin O; LT, *Escherichia coli* heat labile toxin; LTBI, latent TB infection; MCP-3, monocyte chemotactic protein 3; MDR, multi-drug resistant; Mtb, *Mycobacterium tuberculosis;* NK, natural killer; NOX2, NADPH oxidase; Pfo, perfringolysin O; rBCG, recombinant BCG; RD(1-16), region of difference (1-16); ROS, reactive oxygen species; SCID, severe combined immunodeficiency; TB, tuberculosis; T_CM_, central memory T; T_EM_, effector memory T; Th1, T helper cell type 1; T_RM_, resident memory T; XDR, extremely drug resistant.
